# Pre-diagnosis blood DNA methylation profiling of twin pairs discordant for breast cancer points to the importance of environmental risk factors

**DOI:** 10.1186/s13148-024-01767-y

**Published:** 2024-11-18

**Authors:** Hannes Frederik Bode, Liang He, Jacob V. B. Hjelmborg, Jaakko Kaprio, Miina Ollikainen

**Affiliations:** 1 Institute for Molecular Medicine Finland (FIMM), HiLIFE, University of Helsinki, Tukholmankatu 8, 00290 Helsinki, Finland; 2 Research Unit for Epidemiology, Biostatistics and Biodemography, Department of Public Health, University of Southern Denmark, Campusvej 55, 5230 Odense M, Denmark; 3 Minerva Foundation Institute for Medical Research, Tukholmankatu 8, 00290 Helsinki, Finland

**Keywords:** DNA methylation, Breast cancer, Survival analysis, Monozygotic twins, Dizygotic twins

## Abstract

**Background:**

Assessment of breast cancer (BC) risk generally relies on mammography, family history, reproductive history, and genotyping of major mutations. However, assessing the impact of environmental factors, such as lifestyle, health-related behavior, or external exposures, is still challenging. DNA methylation (DNAm), capturing both genetic and environmental effects, presents a promising opportunity. Previous studies have identified associations and predicted the risk of BC using DNAm in blood; however, these studies did not distinguish between genetic and environmental contributions to these DNAm sites. In this study, associations between DNAm and BC are assessed using paired twin models, which control for shared genetic and environmental effects, allowing testing for associations between DNAm and non-shared environmental exposures and behavior.

**Results:**

Pre-diagnosis blood samples of 32 monozygotic (MZ) and 76 dizygotic (DZ) female twin pairs discordant for BC were collected at the mean age of 56.0 years, with the mean age at diagnosis 66.8 years and censoring 75.2 years. We identified 212 CpGs (*p* < 6.4*10^–8^) and 15 DMRs associated with BC risk across all pairs using paired Cox proportional hazard models. All but one of the BC risks associated with CpGs were hypomethylated, and 198/212 CpGs had their DNAm associated with BC risk independent of genetic effects. According to previous literature, at least five of the top CpGs were related to estrogen signaling. Following a comprehensive two-sample Mendelian randomization analysis, we found evidence supporting a dual causal impact of DNAm at cg20145695 (gene body of *NXN*, rs480351) with increased risk for estrogen receptor positive BC and decreased risk for estrogen receptor negative BC.

**Conclusion:**

While causal effects of DNAm on BC risk are rare, most of the identified CpGs associated with the risk of BC appear to be independent of genetic effects. This suggests that DNAm could serve as a valuable biomarker for environmental risk factors for BC, and may offer potential benefits as a complementary tool to current risk assessment procedures.

**Supplementary Information:**

The online version contains supplementary material available at 10.1186/s13148-024-01767-y.

## Background

Breast cancer (BC) risk assessment tools rely on physiological or genetic screening methods such as mammography, family history, and information on reproductive history. In addition to the assessment of major mutations, polygenic risk scores are increasingly used to assess overall genetic risk [1]. However, accurately quantifying the impact of environmental factors, including health-related behaviors and occupational exposures, can be challenging, and these factors are often not included in the risk assessment models [2]. In recent years, DNA methylation (DNAm) has emerged as a promising biomarker for BC risk assessment [3].

BC has been linked to DNAm in blood, as evidenced by specific DNAm sites [4–22] and overall average DNAm levels [3] associating with BC. In addition, the studies conducted by Kresovich, Xiong and Chung and colleagues [7, 19, 23] have shown that blood-derived DNAm can be used to predict an individual's risk of developing BC. This presents an opportunity for blood-derived DNAm to serve as a complementary measure to current standard BC risk assessment tools [24, 25]. In addition, some of the BC risk associating or predicting DNAm sites may be causal to BC [26]. Identification of such causal DNAm biomarkers may contribute to a deeper understanding of the underlying biological mechanisms. However, the earlier work on DNAm biomarkers, whether predictive or causal, and overall BC risk [5, 7, 9, 17, 19, 20, 22] have not differentiated between genetic and environmental risk factors for BC.

Health-related behaviors and environmental exposures are major factors that can increase the risk of BC [27], and many of these factors have been shown to affect DNAm as well, for example, alcohol use [28–30], obesity [31, 32], physical inactivity [33], hormonal exposure [34], and reproduction-related factors [32, 34–36]. In addition, genetic variants, including those linked to BC risk, may affect DNAm.

Twin pairs discordant for disease provides a valuable approach for investigating the impact of environmental factors. Within monozygotic (MZ) twin pairs, the genetic components are fully controlled for as the co-twins are genetically identical at the germ line. Genetic effects are also partially controlled for in the within-pair comparison of dizygotic (DZ) twin pairs, in which the twins share on average 50% of their segregating genetic background. Moreover, the twin design effectively controls for age and shared environmental influences, especially in early life, regardless of whether they are DZ or MZ twin pairs. Although the discordant MZ twin pair design does not capture *de novo* mutations, it dramatically boosts the statistical power and allows robust investigation of the relationship between environmental BC risk and DNAm, while completely controlling for genetic confounding.

In this study, the objective was to investigate the potential of DNAm as a biomarker for environmental BC risk and further to assess causality. To accomplish this, we leveraged a cohort of BC discordant twin pairs from the Finnish Twin Cohort with DNAm data collected prior to BC diagnosis. Then, we aimed to validate the findings by examining an independent BC discordant twin pair dataset from the Danish Twin Study. Finally, we used two-sample MR analysis to find further evidence for causality.

## Methods

### The Finnish Twin Cohort

The Finnish Twin Cohort (FTC), consisting of twins from like-sexed pairs born before 1958, was established in 1975, recruitment was completed by May 1, 1976, and the follow-up period lasted until December 31, 2018. To obtain cancer diagnosis data during the study period, the FTC was linked to the Finnish Cancer Registry. Information on death and emigration was obtained from the Finnish Population Registry. Starting in the 1990s, blood samples were collected from a subset of individuals, and DNA was extracted and stored in the Biobank of the Finnish Institute for Health and Welfare. DNAm data were subsequently generated from these samples.

A group of 108 pairs of female twins who showed discordance for BC at the end of the follow-up period were selected from among all pairs with DNA from the FTC. Of them, 32 pairs were MZ, and 76 pairs were DZ (Table 1). Among the cases, BC was either their first or only cancer diagnosis, while the controls remained cancer-free during the follow-up. The follow-up period was considered to end for cases at the time of diagnosis and for controls either at death (*n* = 18) or latest at the end of the study in 2018 (*n* = 90).

**Table 1:** Description of Finnish Twin Cohort variables used in the survival models

	Monozygotic pairs (n = 32)		Dizygotic pairs (n = 76)	
**DNA methylation platform per pair**				
450K	2		2	
EPIC	30		74	
**Age at sampling in years**				
Mean	57.09		55.54	
SD	10.56		9.64	
Min	38.60		38.12	
Max	79.19		78.91	
Age at sampling in years				
Mean	34.18		33.62	
SD	10.43		9.20	
Min	19.44		18.52	
Max	54.56		60.49	

	Cases^a^	Controls^b^	Cases^a^	Controls^b^
Age at sampling in years				
Mean	66.67	75.55	66.80	75.06
SD	9.53	9.98	9.12	9.27
Min	46.79	60.07	47.53	46.60
Max	87.21	94.76	86.52	91.73
Age at sampling in years				
Mean	9.58	–	11.26	–
SD	6.67	–	6.61	–
Min	0.53	–	0.87	–
Max	21.40	–	23.10	–
Age at sampling in years				
Breast cancer	32 (100%)	–	76 (100%)	–
Death	–	4 (13%)	–	14 (18%)
End of study	–	28 (87%)	–	62 (82%)

^a^Cases refer to the co-twins with breast cancer diagnosis

^b^Controls refer to co-twins that remained cancer-free during the follow-up

Data on epidemiological risk factors for BC were obtained from a health-related questionnaire collected in 1975 (Table 2), while information on the number of children and age at first birth was obtained from the Finnish Population Register for individuals born 1950 onward (Table 2) [37]. The association between these variables and BC discordance was examined using conditional logistic regression for MZ and DZ twin pairs together (Table 2).

**Table 2:** Description and comparison of the available breast cancer risk factors for FTC data

Risk factor	Monozygotic pairs (n = 32)		Dizygotic pairs (n = 76)		All pairs (n = 108)	
	Cases	Controls	Cases	Controls	OR (95%CI)	p-value
**Oral contraceptive use** ^a^						
Never	18 (67%)	19 (63%)	36 (48%)	40 (55%)	1 (reference)	
Ever	9 (33%)	11 (37%)	39 (52%)	33 (45%)	1.45 (0.68, 3.13)	0.34
No data	5	2	1	3		
**Age at first birth in years** ^b^						
Mean	26.69	26.55	25.48	25.39	1.01 (0.91, 1.11)^c^	0.90
SD	5.96	5.15	4.62	5.34		
No data, incl. nulliparous women ^c^	16	12	15	22		
**Number of children incl. nulliparous women** ^b^						
Mean	1.03	1.13	1.76	1.41	1.23 (0.96, 1.58)^c^	0.10
SD	1.40	1.02	1.34	1.43		
No data	2	1	0	0		
Number of nulliparous women	14	11	15	22	0.72 (0.35, 1.47)^d^	0.37
**BMI in kg/m**^**2**^^a^						
Mean	21.72	21.49	22.29	22.78	0.97 (0.86, 1.09)^c^	0.56
SD	2.89	2.60	3.56	3.58		
No data	5	2	3	4		
**Alcohol use in gram ETOH/day** ^a^						
Mean	3.12	2.57	3.90	4.17	0.98 (0.88, 1.09)^c^	0.67
SD	3.51	2.19	3.61	4.12		
No data	5	2	1	3		
**Leisure-time physical activity MET hours/day** ^a^						
Mean	0.76	0.96	1.38	1.09	1.04 (0.89, 1.22)^c^	0.64
SD	0.72	1.22	2.61	1.44		
No data	5	2	7	10		
**Social class** ^a^						
Upper white-collar	1 (4%)	3 (10%)	3 (4%)	4 (5%)	1 (reference)	
Lower white-collar	10 (37%)	14 (47%)	30 (40%)	28 (38%)	1.64 (0.39, 6.87)	0.50
Skilled workers	6 (22%)	5 (17%)	22 (29%)	22 (30%)	1.90 (0.38, 9.65)	0.44
Unskilled workers	3 (11%)	2 (7%)	9 (12%)	4 (5%)	4.21 (0.63, 28.09)	0.14
Farmers	0	1 (4%)	3 (4%)	8 (11%)	0.51 (0.06, 4,32)	0.53
Other (students, retired, unknown)	7 (26%)	5 (17%)	8 (11%)	7 (9%)	2.69 (0.36, 20.39)	0.34
No data	5	2	1	3		
**Education** ^a^						
Primary school or less	11 (41%)	14 (47%)	45 (60%)	44 (60%)	1 (reference)	
Middle school	8 (30%)	6 (20%)	12 (16%)	14 (19%)	1.14 (0.43, 3.07)	0.80
High school graduate	8 (30%)	10 (33%)	17 (23%)	15 (20%)	1.09 (0.29, 4.04)	0.90
Other	0	0	1 (1%)	0	-	
No data	5	2	1	3		
**Relationship status** ^a^						
Not in relationship (single, divorced, widowed)	15 (56%)	10 (33%)	24 (32%)	27 (37%)	1 (reference)	
In relationship (married or unmarried)	12 (44%)	20 (67%)	51 (68%)	46 (63%)	0.89 (0.45, 1.74)	0.73
No data	5	2	1	3		

^a^Based on 1975 questionnaire

^b^Based on data from the Finnish Population Registry

^c^Per 1 increase in unit respectively

^d^Compared to non-nulliparous

MET: Metabolic equivalent is a standardized measure for physical activity, as explained in Jetté et al., 1990. One MET equal to 210 ml O2/kg/hour and represents the oxygen consumption while sitting at rest. [38]

### The Danish Twin Study

The Danish sample is selected from two Danish twin cohorts in the Danish Twin Study (DTS), including the Longitudinal Study of Aging in Danish Twins (LSADT) study and the Middle Age Danish Twins (MADT) study. Details about these twin cohorts are described previously in [39, 40]. Only included those twins that have both DNAm (1180 samples) and the information about BC diagnosis were included, which was retrieved through the link between the twin registry and the cancer registry in the NorTwinCan database [41]. Eighty-six twins in LSADT were measured twice in 1997 and 2007, respectively, for their DNAm, and only the early measurement in 1997 were included to increase the sample size for the analysis of pre-diagnosis DNAm. Among these twins, 11 twin pairs (eight MZ and three DZ pairs) that are discordant for BC and of whom the methylation was measured prior to the diagnosis were included in the analysis. The end of follow-up was defined the same way as for the Finnish dataset.

### DNA methylation data

Among the 108 twin pairs from the FTC, DNAm was measured for four pairs using the Illumina Infinium HumanMethylation450 (450K) platform and for 104 pairs the Illumina Infinium MethylationEPIC (EPIC) platform (Illumina, San Diego, CA, USA), (Table 1). Twins in a pair shared the same platform technology and were sampled and processed at the same time. Preprocessing of the DNAm data was done using the *meffil* R package [42]. Sample quality was assessed based on the following criteria: (1) mean difference between X and Y (technical noise in female samples) chromosome signals was less than -2, (2) mean methylated signal did not deviate from the regression line (mean methylated signal linear regressed over mean unmethylated signal) by more than three standard deviations, (3) sample was not an outlier based on Illumina control probes, (4) percentage of probes with only background signal was less than 20, and (5) percentage of probes with less than three beads was less than 20. All samples that met all the above criteria passed the quality control. In addition, ambiguous mapping and poor-quality probes based on Zhou et al. [43] and probes binding to sex chromosomes were removed to address ambiguity of the DNAm signal based on X-chromosome inactivation [44, 45]. Following the quality control, the DNAm data underwent preprocessing in two separate batches due to use of two different types of microarray platforms, EPIC and 450K.

Following quality control, the DNAm data were preprocessed in two separate sets due to the use of different microarray platforms (EPIC and 450K). The first set combined data from both platforms, focusing on CpG sites shared between them. This increased the sample size, given the limited availability of 450K samples. However, this initial dataset excluded EPIC-specific CpG sites. To address this, we performed a second preprocessing step exclusively on the EPIC samples. In this second set, all CpG sites (including EPIC-specific and shared sites) were included, as shared probes contain relevant reference probes for functional normalization. For the subsequent analysis, we utilized the CpG sites shared between EPIC and 450K from the first set of samples, while adding the EPIC-specific CpG sites from the second set.

The preprocessing was performed by functional normalization using the first 15 principal components of the control probes, to eliminate unwanted technical variation using the *meffil* R package [42]. Additionally, to reduce probe bias, beta-mixture quantile normalization [46] using the *wateRmelon* R package [47] was applied. Next, the CpG probes exclusively present on the EPIC platform were merged with the set of probes common between the EPIC and 450K platforms. Finally, the beta values were scaled based on the standard deviation of each CpG probe across all samples. Standardizing the predictor variables allow us to directly compare the standardized log10(HR) across the CpG probes [48]. For the annotation of the CpG sites, the latest version of the Illumina Infinium Methylation EPIC manifest v1.0 B5 was used (Illumina, San Diego, CA, USA).

After performing quality control and preprocessing, a total of 52,333 probes were removed due to their insufficient quality, and 9918 probes were removed due to their binding to sex chromosomes. A final set of 778,861 probes were retained, consisting of 336,849 probes (43%) shared by the 450K and EPIC data and 442,012 probes exclusive to the EPIC platform.

In the DTS data, DNAm was measured from the buffy coat samples stored at − 80 °C in 24 h after the blood was collected using the 450K platform (Illumina, San Diego, CA, USA). Quality control for sample and probe exclusion was conducted with the *MethylAid* [49] and *Minfi* [50] R packages. More detailed steps of quality control and criteria for excluding samples and probes are described in the previous study [51]. After further excluding any CpG sites that had a missing rate of > 10% across the whole 1180 samples, 451,471 CpG sites remained in the survival analysis.

### Survival modeling

To investigate the association between DNAm and BC risk, a survival analysis was conducted using Cox proportional hazard regression models in the R package *survival* [52]. To ensure that all CpG sites meet the model assumptions, the proportional hazard assumption for the within-pair difference in methylation beta value was examined by Schoenfeld residuals and tested using the *cox.zph()* function for the significant CpG sites. For the analysis, four different Cox proportional hazard models were applied. The equations for all four models are the same, only the cohorts tested with these models different, for example, MZ twin pairs, DZ twin pairs, MZ and DZ twin pairs together, and twin pairs from the DTS (Eq. 1).

1 $$survival\; term\; \left(age\; at\; censoring|BC\; status\right) \sim withinpair\; methylation\; difference + pairwise\; mean\; methylation\; + frailty\; term (pair\; identifier)$$

The survival term in this model refers to the survival outcome observed during the follow-up period. To adjust for the methylation levels specific to each twin pair, the mean beta values for each pair were included as an explanatory variable. The pair identifier is considered as a random effect. Twin-based studies inherently control for genetic and environmental factors that are shared between the twins in a pair. In the current study, the within-pair comparisons further control for other potential confounders such as chip platform and slide, age at entry, and age at sampling.

The discovery cohort involved 108 twin pairs that were sampled before the onset of BC diagnosis (Model 1). Using Bonferroni method for multiple testing correction, *p*-values < 6.4*10^–8^ were considered significant. Results with p-values passing the Benjamini–Hochberg false discovery rate *p* < 0.05, but not passing the Bonferroni correction, are considered marginally significant, and reported in the supplementary materials. The statistically significant CpG sites were followed up in zygosity-specific analyses of MZ pairs (*n* = 32, Model 2) and DZ pairs (*n* = 76, Model 3), to assess the role of genetic versus environmental effects on the significant methylation sites. CpG sites with the same effect direction compared with Model 1 and p < 0.05 were considered as validated. To test the robustness of the finding, we performed a post hoc power analysis on Model 1 (MZ + DZ) using *average.power.coxph()* function in the R package *FDRsamplesize2* [53] at alpha = 6.4*10^–8^. Frequencies of genomic regions in the results were analyzed using Fisher's exact test.

To validate our findings from the FTC samples, we analyzed a second, independent dataset derived from the DTS cohort. This dataset consisted of 11 MZ twin pairs discordant for BC. To replicate our findings in this independent DNAm sample, we fitted a survival model identical to Model 2, as it exclusively includes MZ twin pairs. We refer to this model as Model 2R to indicate its role in replication. The CpG sites were considered as replicated when the direction of the effect was the same as in Model 1 and *p*-value < 0.05.

### Sensitivity analyses

The E-value offers a valuable tool for assessing the robustness of observational studies, by quantifying the strength of unmeasured confounding required to fully explain away an observed association. A large E-value indicates that substantial unmeasured confounding would be necessary to explain away the effect, suggesting a more robust association. Conversely, a small E-value implies that minimal unmeasured confounding could explain the effect, raising concerns about the validity of the association. To evaluate the possible effect of unmeasured confounding variables on the association between DNAm and survival outcomes, E-values were computed for each significant CpG site using the HR of such in the *EValue* R package [54, 55]. E-values for HR > 1 follow Eq. 2 [48]:

2 $$Evalue=1/HR+sqrt(1/HR*\left(1/HR-1\right))$$

E-values for HR < 1 follow Eq. 3 [48]:

3 $$Evalue=HR+sqrt(HR*\left(HR-1\right))$$

### DMR analysis

Differentially methylated regions (DMRs) were identified using the ipDMR() function from the ENmix R package [56]. This function initially identifies pairs of neighboring CpG sites within a 500 base pair window. For each pair, a combined p-value is calculated based on the individual p-values of the intervening CpG sites, obtained from Model 1 [for details see Xu et al. [56]]. Significant CpG site pairs (Benjamini–Hochberg FDR < 0.001, similar as in Petroff et al. ([57]) and Adams et al. ([58])) are then merged into broader regions if they are separated by less than 500 base pairs. New p-values for the association between each region and the survival outcome are calculated using the p-values derived Model 1 for the CpG sites contained in each region [for details see Xu et al. [56]].

A DMR was considered significant if it contained at least three CpG sites, exhibited a consistent direction of methylation association with the survival outcome, and passed Benjamini–Hochberg FDR < 0.001. For each significant region, we report the mean HR and the mean within-pair difference in scaled beta values. These metrics provide a summary of the methylation values associated with the survival outcome within each region.

### Mendelian randomization

To test whether DNAm at the identified CpG sites is causal for BC risk, a two-sample Mendelian randomization (MR) analysis was performed. We established valid genetic instrumental variables (IVs) for the BC–associated DNAm from Model 1, with the following criteria: The identified SNP to be used as IV 1) associates with DNAm at give CpG site of interest (associated with BC under Model 1) with genome-wide significance, 2) does not directly associate with breast cancer, and 3) does not associate with confounders of BC risk or DNAm. First, for the 212 BC–associated CpG sites (Model 1) 18 cis-meQTL SNPs with genome-wide significance (p < 6.9*10^–8^) were identified using the *MeQTL EPIC Database* (https://epicmeqtl.kcl.ac.uk/) [59].

Second, summary statistics from two BC genome-wide association studies (GWAS) were used to eliminate any IVs that directly associate with BC. The first set of summary statistics was derived from the University of Bristol Integrative Epidemiology Unit’s GWAS project (https://gwas.mrcieu.ac.uk/) [60], comprising 212,402 female individuals (6.53% cases) from the UK Biobank (UKBB) made publicly available under a non–commercial government license [61], and the other set was from the FinnGen study release R9 [62] under access rights received on 26th September 2023, encompassing 222,080 female individuals (9.27% cases). Among these, GWAS summary statistics were available for 13 and 12 out of the 18 meQTL SNPs, respectively, and none of these exhibited statistically significant genome-wide association with BC. Additionally, none of the 12 meQTL SNPs available in the FinnGen estrogen receptor positive (ER +) BC GWAS on 213,307 female individuals (5.66% cases) and estrogen receptor negative (ER-) BC GWAS on 209,695 female individuals (4.03% cases) had genome-wide significant association with these BC subtypes.

Third, to eliminate IVs that associate with potential confounders, a phenome-wide association analysis was performed using the GWAS catalog (https://www.ebi.ac.uk/gwas/) [63]. No significant association between any of the IVs and potential confounders was identified. Only rs10228118 associated with height (*p* < 10^–11^), which is a risk factor for BC [64]. However, since height is not an exogenous risk factor for BC and did not differ in cases and control (paired t-test, *p* = 0.66) in the BC discordant pairs, we disregarded this association.

To assess the relationship between exposure (DNAm at the given CpG site) and the outcome (risk for BC), Wald’s ratio testing was employed for each individual SNP (Supplementary Fig. 1), which estimated the causal effect of DNAm on BC risk at the given CpG site. Subsequently, MR-Egger analysis was performed to test pleotropic effects on BC risk across the identified IVs. To test for the directionality from DNAm to BC (BC overall, ER + and ER-BC) risk the MR Steiger test was performed [65]. An association was considered significant if both the Wald’s ratio test and the MR Steiger had nominal *p*-values < 0.05 and the MR Steiger test indicated directionality from DNAm on BC risk. The MR analysis was carried out using the R package *TwoSampleMR* [66].

## Results

### DNAm methylation difference associates with breast cancer in discordant twin pairs

Altogether 108 BC discordant FTC twin pairs (*n* = 32 MZ and *n* = 76 DZ pairs) sampled prior to BC diagnosis were used as the discovery sample (Tables 1 and 2). The average age of study entry was 33.8 years (SD = 9.5 years), and the average age for blood sample collection was 56.0 years (SD = 9.9 years). For cases, the age at diagnosis was on average 66.8 years (SD = 6.6 years), and for controls, the end of follow-up was at an average age of 75.2 years (SD = 9.5 years). The mean time between blood sampling and diagnosis was 10.8 years (SD = 6.7). The association between these variables and BC discordance was examined using conditional logistic regression for MZ and DZ twin pairs together. None of these variables showed a significant association with BC discordance (Table 2).

**Fig. 1:**
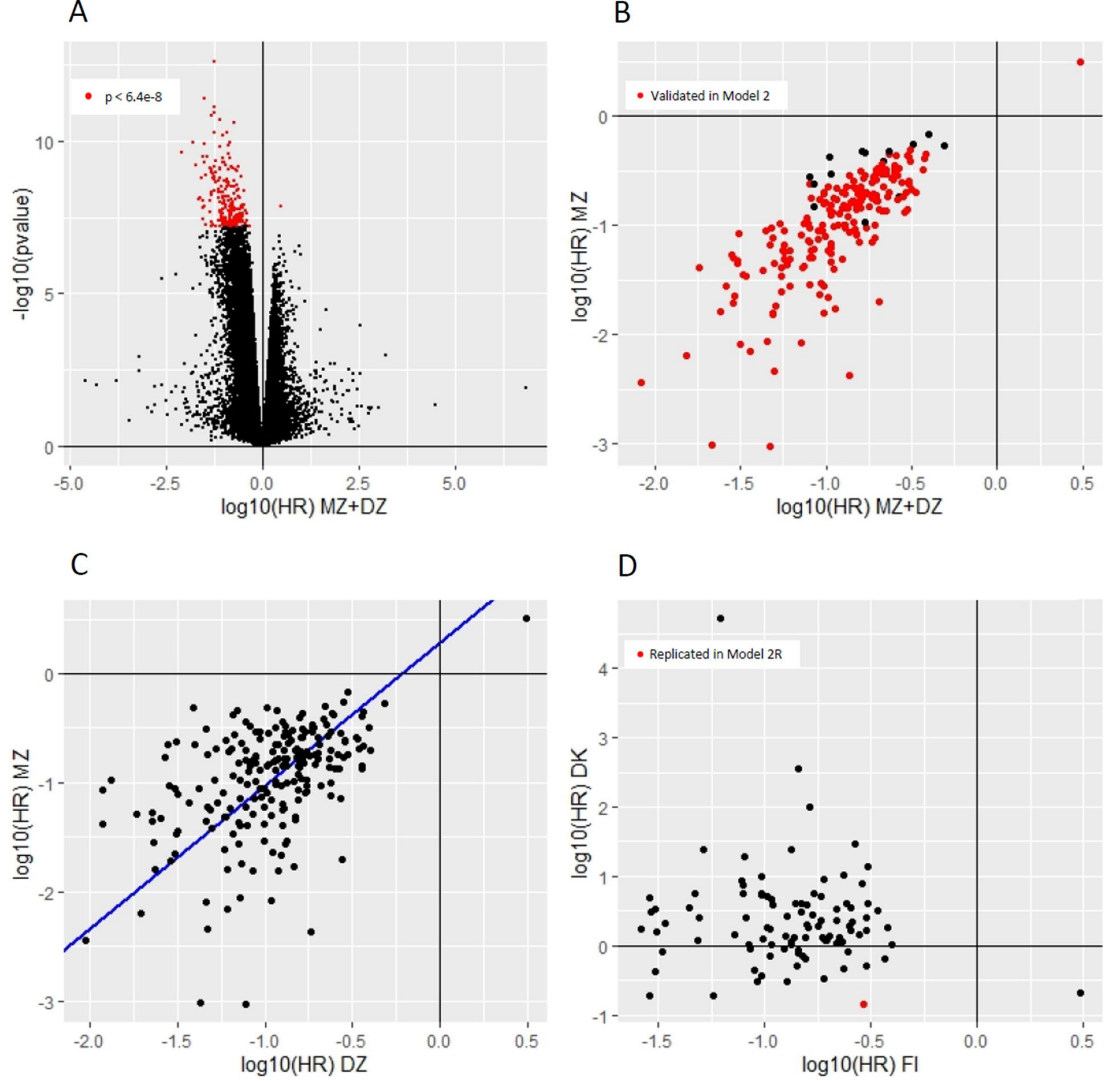
Results on the survival modeling for individual CpG sites associated with breast cancer**; A** Volcano plot of Model 1 (MZ + DZ) with CpG sites significantly associated with breast cancer marked in red; **B** Comparison between Model 1 (MZ + DZ) and Model 2 (MZ) with significant CpG sites from Model 1 validated in Model 2 marked in red; **C** Comparison between Model 2 (MZ) and Model 3 (DZ) with a regression line in blue (regression coefficient = 1.31, p = 0.001); **D** Comparison between Model 1 (MZ + DZ, Finnish data) and Model 2R (MZ + DZ, Danish data) with replicated CpG site in red

The first survival model (Model 1) included MZ and DZ pairs and matched for familial effects. This discovery analysis resulted in 212 CpG sites significantly associated (*p* < 6.4*10^–8^) with future BC (Fig. 1A, and Supplementary Table 1). Among these CpG sites, all except one (cg00550725, in *FAM82B*, more commonly known as *RMDN1*) showed negative association, as indicated by Hazard Ratio (HR) below one, implying that lower DNAm levels associated with higher hazards of BC. The BC-associated hypomethylated CpG sites had HRs ranging from 0.01 to 0.49, while the hypermethylated CpG site had an HR of 3.07. *TDRD1* was the only gene with two significant BC-associated CpG sites (cg14779973 and cg27547703). While most of the significant CpG sites exhibited strong statistical power (mean power = 0.97, SD = 0.11) in Model 1, eight of the 212 sites had power below 0.80 (Supplementary Table 1). Further, all sites that passed a Benjamini–Hochberg FDR < 0.05 in Model 1 are listed in Supplementary Table 4.

A sensitivity analysis was conducted by calculating E-values for the 212 significant CpG sites in Model 1 (Supplementary Table 1). The E-values were high for all CpG sites, indicating that unknown or unmeasured covariates are unlikely to account for the association between DNA methylation at these CpG sites and BC.

In addition to the 212 individual CpG sites, 15 DMRs were significantly associated with BC in Model 1 (Supplementary Table 2). Among these, three DMRs (in genes *SCMH1*, *PXDNL*, and *GNAS*, all relevant for BC biology) contain CpG sites that were also significant as single hits in Model 1. Out of the 15 DMRs, 14 have lower DNAm (average HR < 1) in the BC diagnosed twin compared with their co-twin.

### Environmental breast cancer risk associates with breast cancer-related DNA methylation

To explore whether the 212 BC-associated CpG sites are likely due to environmental effects, we performed within-pair analysis including only MZ twin pairs, which rules out the potential genetic confounding in the observed associations (Model 2). Altogether 198 CpG sites (93%) showed the same effect direction and met nominal significance (*p* < 0.05) for association with BC (Supplementary Table 1, Fig. 1B).

We next assessed the associations in Model 3 containing DZ twin pairs only, which resulted in all the 212 CpG sites having the same effect direction as in Model 1 (MZ + DZ twin pairs) and Model 2 (MZ twin pairs only, independent of germline genetics), and meeting nominal significance (*p* < 0.05) for the association with BC (Supplementary Table 1). However, on average the 212 CpG sites had higher effect sizes in the Model 2 compared with Model 3 (beta = 1.31, *p* = 0.001), suggesting that a higher level of genetic matching results in higher effect size (Fig. 1C).

### Breast cancer-associated DNA methylation pattern is not replicated in an independent cohort

We aimed at replicating the findings from the FTC in the DTS containing 11 twin pairs discordant for breast cancer (eight MZ and three DZ pairs). The mean age at the diagnosis of these 11 twin pairs was 78.1 (SD = 9.6 years), and the mean age at the DNAm profiling was 71.3 (SD = 6.6 years). The DTS datasets included 98 out of the 212 CpG sites identified in the discovery analysis performed in FTC Model 1. The remaining sites were not present in the DTS data, which were generated using the smaller 450K methylation platform. Out of these 98 CpG sites, 22 had the same effect direction in the DTS (Model2R) as in the FTC data (Model 1 and Model 2). However, only one of these CpG sites (cg16376218, within *SLC25A39*) met nominal significance (*p* < 0.05) for the association with BC (Supplementary Table 1, Fig. 1D).

### Breast cancer-associated DNA methylation exhibit distinct genomic distribution

**Fig. 2:**
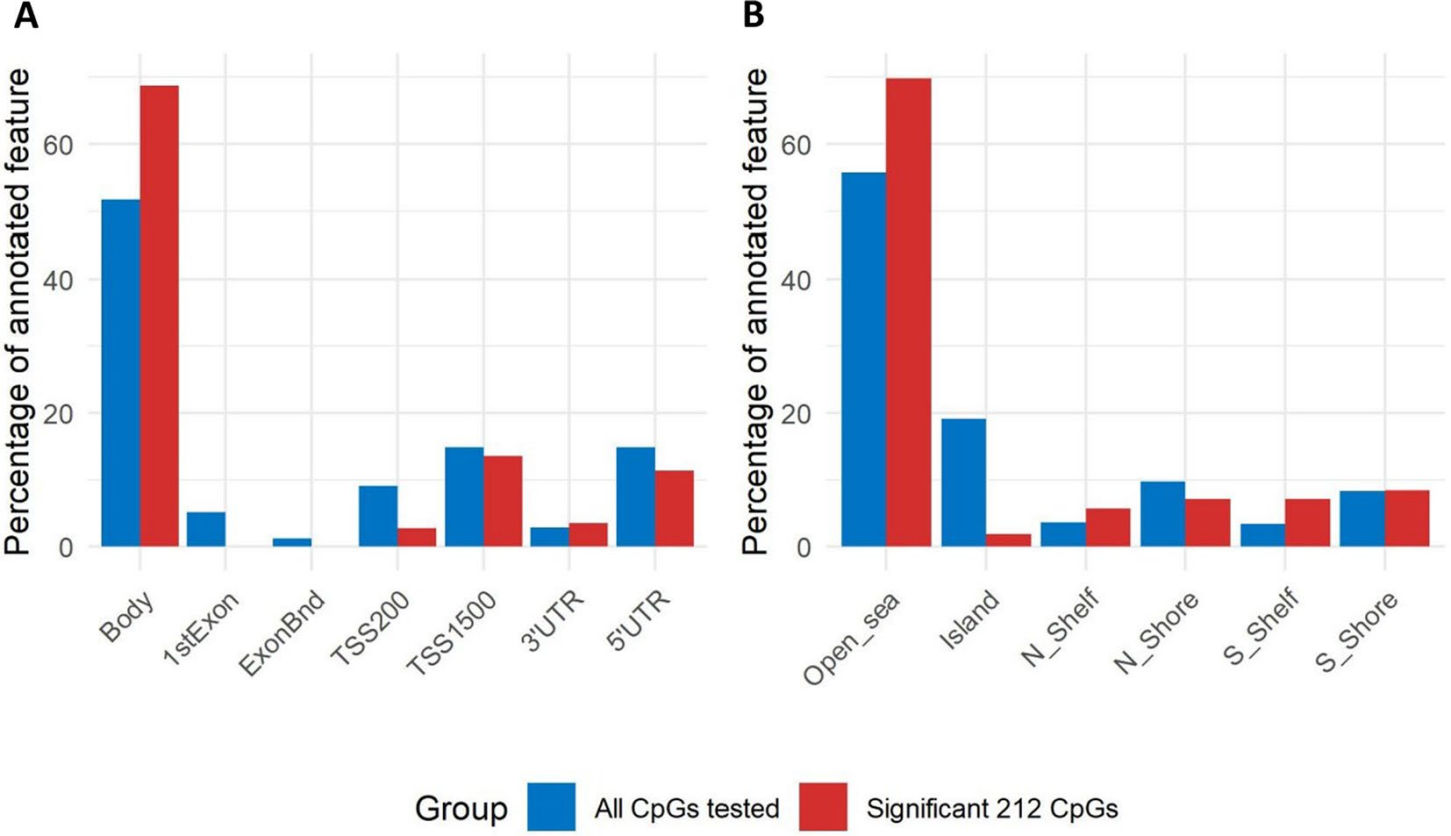
Comparison of the distribution of annotated features for 212 CpG sites associated with BC under Model 1 (MZ + DZ) to all tested CpG sites. Panel A shows differences in the presence of gene regions, while Panel B shows differences in the presence of CpG island features

To identify overrepresented features in our data, we compared the distribution of the significant CpG sites from Model 1 (MZ + DZ) to all tested CpG sites based on the Illumina Infinium Methylation EPIC manifest v1.0 B5. We observed distinct differences in the genomic distribution in regard to both gene context and CpG density of the BC-associated CpG sites compared to all CpG sites measured. Specifically, the significant sites were overrepresented within gene bodies (68.7% observed vs. 51.7% expected, *p* < 1.3*10–9) but underrepresented in regulatory regions such as first exons (0.0% observed vs. 5.2% expected, *p* < 8.1*10^–8^), exon binding sites (0.0% observed vs. 1.2% expected, *p* = 0.04), and transcription start sites (16.4% observed vs. 25.0% expected, *p* = 0.002) (Fig. 2A). Further, the core regions of CpG islands (1.9% observed vs. 19.1% expected, *p* < 1.4*10^–14^) were underrepresented, while open sea regions (69.8% observed vs. 55.8% expected, *p* < 3.1*10^–5^) and south shelves (7.1% observed vs. 3.4% expected, *p* = 0.006) were overrepresented (Fig. 2B).

### Evidence for a single CpG site being causal for BC risk

Our study design, DNAm measured before BC diagnosis and the within-pair comparisons ruling out confounding by shared genetic and environmental effects, suggests that the observed BC–associated DNAm patterns precede BC. We next aimed at assessing if there is additional evidence for direct causation by performing a two-sample MR analysis. Altogether 12 genome-wide significant meQTLs met the criteria (see Methods) for IVs and were used in the two-sample MR to test for causality for BC in general, and for ER + and ER- subtypes. The MR analysis was performed using Wald’s Ratio testing for individual IVs. Egger regression analysis across all available IVs revealed no pleiotropy, and the Steiger MR test showed that directionality went from DNAm to BC risk for all IVs.

No causal effects were observed for BC risk in general for the 12 CpG sites (Supplementary Table 3). Using the 11 out of 12 IVs available for ER + and ER-BC (Supplementary Table 3), DNAm at cg20145695 (*NXN*, rs480351) only showed significant causal effect. Higher methylation increased the risk for ER + BC (OR_ER+_ = 1.51 (CI95 = 1.02–2.24), *p* = 0.04) and decreased the risk for ER-BC (OR_ER-_ = 0.72 (CI95 = 0.52–0.98), *p* = 0.04), showing that DNAm at this site has effects opposing each other depending on the BC subtype. (Supplementary Table 3).

## Discussion

Here, we studied the association between BC risk and DNAm in blood samples taken prior to cancer diagnosis. We found that 212 CpG sites and 15 DMRs in blood were associated with the risk of BC using a discordant twin study design, matched for familial confounders. These CpG sites were enriched in gene bodies and open sea regions but depleted from CpG island cores. Among the 15 DMRs we identified, three contain CpG sites that also associate individually with BC risk. These CpG sites are located in the genes *SCMH1*, *PXDNL*, and *GNAS*. We validated the majority of the BC risk associated CpG sites (198/212) within MZ pairs, and with significantly higher effect sizes than within DZ pairs, suggesting that these 198 CpG sites associate with BC independent of genetic factors and are likely attributed to environmental BC risk. Only one of these significant sites (cg16376218 in *SLC25A39*) replicated in the Danish Twin Study.

Our study validated one previously observed BC-associated CpG site (cg21769444, *NUDT3*) [21]. *NUDT3*-associated long noncoding RNA, NUDT3-AS4, appears to promote cell growth in BC. It acts as a sponge for microRNA miR-99 s, competing with AKT1/mTOR mRNAs for binding. This competition prevents miR-99 s from degrading AKT1/mTOR mRNAs, leading to increased expression of AKT1 and mTOR proteins. Overexpression of these proteins is linked to the abnormal PI3K/AKT/mTOR pathway contributing to increased cancer cell proliferation, a common feature in many cancers including BC [67]. The dysregulation caused by NUDT3-AS4 via DNAm may therefore contribute to increased BC risk.

Most of the identified DNAm sites were associated with BC risk independent of genetic effects, and may reflect within-pair differences in exposures to environmental BC risk factors, such as alcohol consumption, exposure to sex hormones [68], and risk factors previously identified in twin studies such as age at first birth, number of children and age at menopause [69], and BMI [70]. Identification of genes related to estrogen signaling (*TDRD1* [71], *SCMH1* [34], *PXDNL* [72], *GNAS* [73], and *RMDN1* [74]) suggests that within-pair differences in hormonal exposures may have resulted in the observed within-pair differences in DNAm patterns associated with future BC diagnosis. In particular, identification of BC-associated DNAm in *SCMH1*, which has been associated with lifetime exposure to sex hormones [34], reinforces this. However, based on the available phenotype data, we could not demonstrate clear differences in hormone exposure (nor other risk factors) between the twin with BC and her healthy sister. In addition to the factors included in our analysis, several other factors not directly assessed in this study could contribute differences in hormone exposure. These factors include exposure to endocrine-disrupting chemicals, such as bisphenol A, phthalates [75], but also exposure to stress [76] or shift-work [77], can increase BC risk likely through hormonal disruption. Further, it is important to recognize that, for example, hormonal exposure due to environmental factors may not be determined by individual factors alone, but rather by the complex combination of multiple factors over time [65], and thereby would be difficult to address using the given phenotype data. There may also be other contributing risk factors, which we have not been able to assess. Investigation of the role of individual risk factors contributing to DNAm changes prior to disease onset requires larger and targeted studies.

A search of the EWAS Catalog (http://www.ewascatalog.org/) [78] and EWAS-Atlas (https://ngdc.cncb.ac.cn/ewas/browse) [79] was conducted to identify previously published EWAS summary statistics on BC risk factors. This was done to assess whether the 212 CpG sites identified in this study had already been described as associated with findings in other research. However, no overlap was found between the results of this study and the data available in these two databases.

All of the observed DNAm differences within the pairs discordant for BC have originated prior to BC diagnosis, and they are mostly driven by environmental effects. We were able to test for direct causation for only 12 of the 212 significant sites as for the rest of the significant CpG sites we could not identify suitable IVs for the MR analysis. For the available IVs only in one CpG site (cg20145695) DNAm could be causally linked to BC. DNAm at cg20145695 increased the risk for ER + BC and decreased the risk for ER-BC. This CpG site is located within the gene *NXN* coding for nucleoredoxin. Nucleoredoxin interacts with protein flightless-1 homolog [80], which has been demonstrated to enhance genome accessibility at estrogen receptor targets in MCF7 BC cell lines [81]. Changes in *NXN* expression through methylation at cg20145695 could modulate the activity of protein flightless-1 homolog, which impact the accessibility of estrogen targets on the genome. Thereby, the cell's sensitivity to estrogen signaling could potentially be impacted, distinguishing ER + from ER-BC, and may explain our finding on *NXN* methylation resulting in opposite risks for ER + and ER-BC. However, while our results suggest a potential link between DNAm and BC risk, additional research through functional studies is necessary to establish causality and to understand the biological mechanisms involved.

Notable strengths of this study were embedded into the study design. The within-pair comparisons account for both known and unknown factors that could confound DNAm analysis, including genotype, age, and familial effects shared by the co-twins that differ for BC diagnosis. The DNAm data used for analysis were collected on average 11 years prior to BC diagnosis, which effectively minimizes the potential confounding of BC as a disease and its treatment on the DNAm increasing the power to detect true BC risk-related associations. However, we saw a limited reproducibility of our findings in the DTS. The DTS is characterized by a considerably smaller sample size, and it thereby likely lacks the statistical power required for robust replication. Further, the FTC DNAm data were derived from whole blood, while the DTS DNAm data were obtained from buffy coat. While differences in cell type composition between these sample types could potentially be reflected in the observed DNAm patterns, previous studies have demonstrated that whole blood and buffy coat samples can serve as reasonable proxies for each other [82, 83]. Another limitation of this study is the relatively small sample size of FTC; however, the current study is the largest conducted so far for twin pairs discordant for BC. While most CpG sites exhibited sufficient statistical power, a small number did not reach the critical power threshold of 0.80. Nevertheless, the overall mean power across the significant sites in Model 1 was high.

Our results showed causal evidence for a single CpG site with its methylation causing the opposite risk effect for ER + and ER-BC. This is in line with the notion that the risk for distinct BC subtypes, especially the hormone receptor positive versus hormone receptor negative BC, are impacted by varying environmental risk factors [84]. As the blood samples for DNAm were collected on average 11 years prior to diagnosis, this timespan might be too long for observing strong causal effects of DNAm on BC risk. An alternative explanation could be that DNAm is associated with the risk of the disease through confounding arising from exposures that simultaneously influence both BC risk and DNAm, albeit through different pathways. While understanding the relationship between DNAm and BC risk is crucial for a broader understanding of disease etiology, it is important to note that this limitation does not impact the utility of DNAm as a biomarker for BC risk.

## Conclusion

This study demonstrated the presence of a BC-associated DNAm patterns in blood, on average 11 years before the actual BC diagnosis. These patterns were independent of familial factors and likely due to individual environmental exposures. Furthermore, these findings suggest that DNAm could be a promising addition to BC risk assessment toolset for identifying individuals who have a higher likelihood of developing BC. Importantly, our study reveals DNAm at a single CpG site to simultaneously increase the risk for ER + and decrease the risk for ER-BC. Future studies in larger prospective cohorts are warranted to clarify which environmental factors are most relevant to BC risk mediated by DNAm.

## Supplementary Information

Additional file 1.

Additional file 2.

## Data Availability

The Finnish Twin Cohort phenotype and methylation data utilized in the current study are deposited in the Biobank of the Finnish Institute for Health and Welfare, Helsinki, Finland. Access to Finnish Cancer Registry data can be applied for as part of the Biobank access procedures. These data are available for use by qualified researchers through a standardized application procedure (https://thl.fi/en/statistics-and-data/data-and-services/research-use-and-data-permits). Survey data and biological samples for the Danish Twin Study are available at the Danish Twin Registry at the Department of Public Health, University of Southern Denmark, Odense, Denmark (https://www.sdu.dk/en/om_sdu/institutter_centre‌‌‌/ist_sundhedstjenesteforsk‍‌/‌‌‌‌‌centre/dtr/researcher). For more information on the Danish Twin Registry, please refer to Pedersen et al. 2019 [39].

## Abbreviations

BC: Breast cancer
DTS: Danish Twin Study
DZ: Dizygotic
EPIC: Illumina Infinium MethylationEPIC BeadChip
ER + BC: Estrogen receptor positive breast cancer
ER-BC: Estrogen receptor negative breast cancer
EWAS: Epigenome-wide association study
FDR: False discovery rate
FTC: Finnish Twin Cohort Study
GWAS: Genome-wide association study
HR: Hazard Ratio
IV: Instrumental variable
LSADT: Longitudinal study of aging in Danish Twins Study
MADT: Middle Age Danish Twins Study
MR: Mendelian randomization
MZ: Monozygotic
OR: Odds Ratio
UKBB: UK Biobank
450K: Infinium HumanMethylation450 BeadChip

## References

[1] Roberts E, Howell S, Evans DG. Polygenic risk scores and breast cancer risk prediction. Breast Off J Eur Soc Mastology. 2023;67:71. 10.1016/j.breast.2023.01.003 PMC9982311 36646003

[2] Nwanaji-Enwerem JC, Colicino E. DNA Methylation-Based Biomarkers of Environmental Exposures for Human Population Studies. Curr Environ Heal reports. 2020;7:121–8. 10.1007/s40572-020-00269-2 PMC12910356 32062850

[3] Tang Q, Cheng J, Cao X, Surowy H, Burwinkel B. Blood-based DNA methylation as biomarker for breast cancer: a systematic review. Clin Epigenetics. 2016. 10.1186/S13148-016-0282-6. 27895805 10.1186/s13148-016-0282-6 PMC5109688

[4] Johansson A, Flanagan JM. Epigenome-wide association studies for breast cancer risk and risk factors. Trends Cancer Res. 2017;12:19. 28955137 PMC5612397

[5] Massi MC, Dominoni L, Ieva F, Fiorito G. A Deep Survival EWAS approach estimating risk profile based on pre-diagnostic DNA methylation: An application to breast cancer time to diagnosis. PLoS Comput Biol. 2022. 10.1371/JOURNAL.PCBI.1009959. 36155971 10.1371/journal.pcbi.1009959 PMC9536632

[6] Joo JE, Dowty JG, Milne RL, et al. Heritable DNA methylation marks associated with susceptibility to breast cancer. Nat Commun. 2018. 10.1038/S41467-018-03058-6. 29491469 10.1038/s41467-018-03058-6 PMC5830448

[7] Kresovich JK, Xu Z, O’Brien KM, Shi M, Weinberg CR, Sandler DP, Taylor JA. Blood DNA methylation profiles improve breast cancer prediction. Mol Oncol. 2022;16:42. 34411412 10.1002/1878-0261.13087 PMC8732352

[8] Tuminello S, Zhang Y, Yang L, et al. Global DNA methylation profiles in peripheral blood of WTC-exposed community members with breast cancer. Int J Environ Res Public Heal. 2022;19:5104. 10.3390/ijerph19095104 PMC9105091 35564499

[9] Ennour-Idrissi K, Dragic D, Issa E, Michaud A, Chang SL, Provencher L, Durocher F, Diorio C. DNA methylation and breast cancer risk: an epigenome-wide study of normal breast tissue and blood. Cancers (Basel). 2020;12:1–16. 10.3390/cancers12113088 PMC7690691 33113958

[10] Ho PJ, Dorajoo R, Ivanković I, et al. DNA methylation and breast cancer-associated variants. Breast Cancer Res Treat. 2021;188:713–27. 33768416 10.1007/s10549-021-06185-9

[11] Shenker NS, Polidoro S, van Veldhoven K, Sacerdote C, Ricceri F, Birrell MA, Belvisi MG, Brown R, Vineis P, Flanagan JM. Epigenome-wide association study in the European Prospective Investigation into Cancer and Nutrition (EPIC-Turin) identifies novel genetic loci associated with smoking. Hum Mol Genet. 2013;22:843–51. 23175441 10.1093/hmg/dds488

[12] Yao S, Hu Q, Kerns S, et al. Impact of chemotherapy for breast cancer on leukocyte DNA methylation landscape and cognitive function: a prospective study. Clin Epigenetics. 2019. 10.1186/S13148-019-0641-1. 30867049 10.1186/s13148-019-0641-1 PMC6416954

[13] Wong EM, Southey MC, Terry MB. Integrating DNA methylation measures to improve clinical risk assessment: are we there yet? The case of BRCA1 methylation marks to improve clinical risk assessment of breast cancer. Br J Cancer. 2020;122:1133–40. 32066913 10.1038/s41416-019-0720-2 PMC7156506

[14] Heyn H, Carmona FJ, Gomez A, et al. DNA methylation profiling in breast cancer discordant identical twins identifies DOK7 as novel epigenetic biomarker. Carcinogenesis. 2013;34:102. 23054610 10.1093/carcin/bgs321 PMC3534196

[15] Scott CM, Wong EM, Joo JHE, Dugué PA, Jung CH, O’Callaghan N, Dowty J, Giles GG, Hopper JL, Southey MC. Genome-wide DNA methylation assessment of ‘BRCA1-like’ early-onset breast cancer: Data from the Australian Breast Cancer Family Registry. Exp Mol Pathol. 2018;105:404–10. 30423315 10.1016/j.yexmp.2018.11.006 PMC6289279

[16] Anjum S, Fourkala EO, Zikan M, et al. A BRCA1-mutation associated DNA methylation signature in blood cells predicts sporadic breast cancer incidence and survival. Genome Med. 2014. 10.1186/GM567. 25067956 10.1186/gm567 PMC4110671

[17] Xu Z, Sandler DP, Taylor JA. Blood DNA Methylation and Breast Cancer: A Prospective Case-Cohort Analysis in the Sister Study. J Natl Cancer Inst. 2020;112:87–94. 30989176 10.1093/jnci/djz065 PMC7489106

[18] Yang Y, Wu L, Shu XO, et al. Genetically predicted levels of DNA methylation biomarkers and breast cancer Risk: data from 228 951 women of European Descent. J Natl Cancer Inst. 2020;112:295–304. 31143935 10.1093/jnci/djz109 PMC7073907

[19] Xiong Z, Yang L, Ao J, Yi J, Zouxu X, Zhong W, Feng J, Huang W, Wang X, Shuang Z. A prognostic model for breast cancer based on cancer incidence-related DNA methylation pattern. Front Genet. 2022. 10.3389/FGENE.2021.814480. 35047022 10.3389/fgene.2021.814480 PMC8762114

[20] Xu Z, Bolick SCE, Deroo LA, Weinberg CR, Sandler DP, Taylor JA. Epigenome-wide association study of breast cancer using prospectively collected sister study samples. JNCI J Natl Cancer Inst. 2013;105:694. 23578854 10.1093/jnci/djt045 PMC3653821

[21] Parashar S, Cheishvili D, Mahmood N, Arakelian A, Tanvir I, Khan HA, Kremer R, Mihalcioiu C, Szyf M, Rabbani SA. DNA methylation signatures of breast cancer in peripheral T-cells. BMC Cancer. 2018. 10.1186/S12885-018-4482-7. 29776342 10.1186/s12885-018-4482-7 PMC5960123

[22] Cappetta M, Fernandez L, Brignoni L, Artagaveytia N, Bonilla C, López M, Esteller M, Bertoni B, Berdasco M. Discovery of novel DNA methylation biomarkers for non-invasive sporadic breast cancer detection in the Latino population. Mol Oncol. 2021;15:473–86. 33145876 10.1002/1878-0261.12842 PMC7858097

[23] Chung FFL, Maldonado SG, Nemc A, et al. Buffy coat signatures of breast cancer risk in a prospective cohort study. Clin Epigenetics. 2023. 10.1186/S13148-023-01509-6. 37309009 10.1186/s13148-023-01509-6 PMC10262593

[24] Pashayan N, Pharoah P. Population-based screening in the era of genomics. Per Med. 2012;9:451–5. 22984365 10.2217/pme.12.40 PMC3442228

[25] Garcia-Closas M, Gunsoy NB, Chatterjee N. Combined associations of genetic and environmental risk factors: implications for prevention of breast cancer. J Natl Cancer Inst. 2014. 10.1093/JNCI/DJU305. 25392194 10.1093/jnci/dju305 PMC4271030

[26] Grau-Perez M, Agha G, Pang Y, Bermudez JD, Tellez-Plaza M. Mendelian randomization and the environmental epigenetics of health: a systematic review. Curr Environ Heal reports. 2019;6:38–51. 10.1007/s40572-019-0226-3 30773605

[27] Kashyap D, Pal D, Sharma R, Garg VK, Goel N, Koundal D, Zaguia A, Koundal S, Belay A. Global Increase in Breast Cancer Incidence: Risk Factors and Preventive Measures. Biomed Res Int. 2022. 10.1155/2022/9605439. 35480139 10.1155/2022/9605439 PMC9038417

[28] Varela-Rey M, Woodhoo A, Martinez-Chantar ML, Mato JM, Lu SC. Alcohol, DNA methylation, and cancer. Alcohol Res. 2013;35:25. 24313162 10.35946/arcr.v35.1.04 PMC3860423

[29] Mahna D, Puri S, Sharma S. DNA methylation signatures: Biomarkers of drug and alcohol abuse. Mutat Res Mutat Res. 2018;777:19–28. 10.1016/j.mrrev.2018.06.002 30115428

[30] Dragic D, Chang SL, Ennour-Idrissi K, Durocher F, Severi G, Diorio C. Association between alcohol consumption and DNA methylation in blood: a systematic review of observational studies. Epigenomics. 2022;14:793–810. 35762294 10.2217/epi-2022-0055

[31] Dragic D, Ennour-Idrissi K, Michaud A, Chang SL, Durocher F, Diorio C. Association between BMI and DNA methylation in blood or normal adult breast tissue: a systematic review. Anticancer Res. 2020;40:1797–808. 32234868 10.21873/anticanres.14134

[32] Chen M, Wong EM, Nguyen TL, et al. DNA methylation-based biological age, genome-wide average DNA methylation, and conventional breast cancer risk factors. Sci Rep. 2019. 10.1038/S41598-019-51475-4. 31636290 10.1038/s41598-019-51475-4 PMC6803691

[33] Światowy WJ, Drzewiecka H, Kliber M, Sąsiadek M, Karpiński P, Pławski A, Jagodziński PP. Physical activity and DNA methylation in humans. Int J Mol Sci. 2021. 10.3390/IJMS222312989. 34884790 10.3390/ijms222312989 PMC8657566

[34] Johansson A, Palli D, Masala G, et al. Epigenome-wide association study for lifetime estrogen exposure identifies an epigenetic signature associated with breast cancer risk. Clin Epigenetics. 2019. 10.1186/S13148-019-0664-7. 31039828 10.1186/s13148-019-0664-7 PMC6492393

[35] Levine ME, Lu AT, Chen BH, et al. Menopause accelerates biological aging. Proc Natl Acad Sci U S A. 2016;113:9327–32. 27457926 10.1073/pnas.1604558113 PMC4995944

[36] Kresovich JK, Xu Z, O’Brien KM, Weinberg CR, Sandler DP, Taylor JA. Methylation-based biological age and breast cancer risk. JNCI J Natl Cancer Inst. 2019;111:1051. 30794318 10.1093/jnci/djz020 PMC6792078

[37] Rose RJ, Latvala A, Silventoinen K, Kaprio J. Alcohol consumption at age 18–25 and number of children at a 33-year follow-up: Individual and within-pair analyses of Finnish twins. Alcohol Clin Exp Res. 2022;46:1552–64. 35719054 10.1111/acer.14886 PMC9545724

[38] Jetté M, Sidney K, Blümchen G. Metabolic equivalents (METS) in exercise testing, exercise prescription, and evaluation of functional capacity. Clin Cardiol. 1990;13:555–65. 2204507 10.1002/clc.4960130809

[39] Pedersen DA, Larsen LA, Nygaard M, et al. The Danish twin registry: an updated overview. Twin Res Hum Genet. 2019;22:499. 31544734 10.1017/thg.2019.72 PMC8039015

[40] Skytthe A, Harris JR, Czene K, et al. Cancer incidence and mortality in 260,000 nordic twins with 30,000 prospective cancers. Twin Res Hum Genet. 2019;22:99–107. 31020942 10.1017/thg.2019.10

[41] Harris JR, Hjelmborg J, Adami HO, Czene K, Mucci L, Kaprio J. The Nordic Twin Study on Cancer — NorTwinCan. Twin Res Hum Genet. 2019;22:817–23. 31512575 10.1017/thg.2019.71

[42] Min JL, Hemani G, Smith GD, Relton C, Suderman M. Meffil: efficient normalization and analysis of very large DNA methylation datasets. Bioinformatics. 2018;34:3983–9. 29931280 10.1093/bioinformatics/bty476 PMC6247925

[43] Zhou W, Laird PW, Shen H. Comprehensive characterization, annotation and innovative use of Infinium DNA methylation BeadChip probes. Nucleic Acids Res. 2017;45:e22–e22. 27924034 10.1093/nar/gkw967 PMC5389466

[44] Inkster AM, Wong MT, Matthews AM, Brown CJ, Robinson WP. Who’s afraid of the X? Incorporating the X and Y chromosomes into the analysis of DNA methylation array data. Epigenetics Chromatin. 2023. 10.1186/S13072-022-00477-0. 36609459 10.1186/s13072-022-00477-0 PMC9825011

[45] Feil R, Khosla S. Genomic imprinting in mammals: An interplay between chromatin and DNA methylation? Trends Genet. 1999;15:431–5. 10529801 10.1016/s0168-9525(99)01822-3

[46] Teschendorff AE, Marabita F, Lechner M, Bartlett T, Tegner J, Gomez-Cabrero D, Beck S. A beta-mixture quantile normalization method for correcting probe design bias in Illumina Infinium 450 k DNA methylation data. Bioinformatics. 2013;29:189–96. 23175756 10.1093/bioinformatics/bts680 PMC3546795

[47] Pidsley R, Wong CCY, Volta M, Lunnon K, Mill J. Schalkwyk LC (2013) A data-driven approach to preprocessing Illumina 450K methylation array data. BMC Genomics. 2013;141(14):1–10. 10.1186/1471-2164-14-293 PMC3769145 23631413

[48] Siegel AF. Multiple regression: predicting one variable from several others. Pract Bus Stat. 2012. 10.1016/B978-0-12-385208-3.00012-2.

[49] van Iterson M, Tobi EW, Slieker RC, den Hollander W, Luijk R, Slagboom PE, Heijmans BT. MethylAid: visual and interactive quality control of large Illumina 450k datasets. Bioinformatics. 2014;30:3435–7. 25147358 10.1093/bioinformatics/btu566

[50] Aryee MJ, Jaffe AE, Corrada-Bravo H, Ladd-Acosta C, Feinberg AP, Hansen KD, Irizarry RA. Minfi: a flexible and comprehensive Bioconductor package for the analysis of Infinium DNA methylation microarrays. Bioinformatics. 2014;30:1363–9. 24478339 10.1093/bioinformatics/btu049 PMC4016708

[51] Soerensen M, Hozakowska-Roszkowska DM, Nygaard M, Larsen MJ, Schwämmle V, Christensen K, Christiansen L, Tan Q. A Genome-Wide Integrative Association Study of DNA Methylation and Gene Expression Data and Later Life Cognitive Functioning in Monozygotic Twins. Front Neurosci. 2020;14: 517712. 10.3389/fnins.2020.00233 PMC7160301 32327964

[52] Therneau T (2023) A Package for Survival Analysis in R.

[53] Ni Y, Seffernick AE, Onar-Thomas A, Pounds SB. Computing power and sample size for the false discovery rate in multiple applications. Genes (Basel). 2024. 10.3390/GENES15030344/S1. 38540403 10.3390/genes15030344 PMC10970028

[54] Van Der Weele TJ, Ding P. Sensitivity analysis in observational research: introducing the E-value. Ann Intern Med. 2017;167:268–74. 28693043 10.7326/M16-2607

[55] Korhonen T, Hjelmborg J, Harris JR, Clemmensen S, Adami HO, Kaprio J. Cancer in twin pairs discordant for smoking: the Nordic twin study of cancer. Int J Cancer. 2022;151:33. 35143046 10.1002/ijc.33963 PMC9304125

[56] Xu Z, Xie C, Taylor JA, Niu L. ipDMR: identification of differentially methylated regions with interval P-values. Bioinformatics. 2021;37:711–3. 32805005 10.1093/bioinformatics/btaa732 PMC8248314

[57] Petroff RL, Dolinoy DC, Wang K, et al. Translational toxicoepigenetic Meta-Analyses identify homologous gene DNA methylation reprogramming following developmental phthalate and lead exposure in mouse and human offspring. Environ Int. 2024. 10.1016/J.ENVINT.2024.108575. 38507935 10.1016/j.envint.2024.108575 PMC11463831

[58] Adams C, Nair N, Plant D, et al. Identification of Cell-specific differential DNA methylation associated with methotrexate treatment response in rheumatoid arthritis. Arthritis Rheumatol (Hoboken, NJ). 2023;75:1088–97. 10.1002/art.42464 PMC10313739 36716083

[59] Villicaña S, Castillo-Fernandez J, Hannon E, et al. Genetic impacts on DNA methylation help elucidate regulatory genomic processes. Genome Biol. 2023. 10.1186/S13059-023-03011-X. 37525248 10.1186/s13059-023-03011-x PMC10391992

[60] Burrows K, Bull CJ, Dudding T, Gormley M, Robinson T, Tan V, Yarmolinsky J, Haycock PC (2021) Genome-wide Association Study of Cancer Risk in UK Biobank. 10.5523/bris.1ovaau5sxunp2cv8rcy88688v

[61] Elsworth​ B, Lyon​ M, Alexander​ T, et al (2020) The MRC IEU OpenGWAS data infrastructure. bioRxiv 2020.08.10.244293

[62] Kurki MI, Karjalainen J, Palta P, et al. FinnGen provides genetic insights from a well-phenotyped isolated population. Nature. 2023;613:508. 36653562 10.1038/s41586-022-05473-8 PMC9849126

[63] Sollis E, Mosaku A, Abid A, et al. The NHGRI-EBI GWAS Catalog: knowledgebase and deposition resource. Nucleic Acids Res. 2023;51:D977–85. 36350656 10.1093/nar/gkac1010 PMC9825413

[64] Zhang B, Shu XO, Delahanty RJ, et al. Height and breast cancer risk: evidence from prospective studies and mendelian randomization. JNCI J Natl Cancer Inst. 2015. 10.1093/JNCI/DJV219. 26296642 10.1093/jnci/djv219 PMC4643630

[65] Hemani G, Tilling K, Davey Smith G. Orienting the causal relationship between imprecisely measured traits using GWAS summary data. PLoS Genet. 2017. 10.1371/JOURNAL.PGEN.1007081. 29149188 10.1371/journal.pgen.1007081 PMC5711033

[66] Hemani G, Zheng J, Elsworth B, et al. The MR-Base platform supports systematic causal inference across the human phenome. Elife. 2018. 10.7554/ELIFE.34408. 29846171 10.7554/eLife.34408 PMC5976434

[67] Hao Q, Wang P, Dutta P, et al. Comp34 displays potent preclinical antitumor efficacy in triple-negative breast cancer via inhibition of NUDT3-AS4, a novel oncogenic long noncoding RNA. Cell Death Dis. 2020. 10.1038/S41419-020-03235-W. 33311440 10.1038/s41419-020-03235-w PMC7733521

[68] Obeagu EI, Obeagu GU. Breast cancer: A review of risk factors and diagnosis. Medicine (Baltimore). 2024;103:E36905. 38241592 10.1097/MD.0000000000036905 PMC10798762

[69] Hamilton AS, Mack TM. Puberty and genetic susceptibility to breast cancer in a case-control study in twins. N Engl J Med. 2003;348:2313–22. 12788995 10.1056/NEJMoa021293

[70] Lundqvist E, Kaprio J, Verkasalo PK, Pukkala E, Koskenvuo M, Söderberg KC, Feychting M. Co-twin control and cohort analyses of body mass index and height in relation to breast, prostate, ovarian, corpus uteri, colon and rectal cancer among Swedish and Finnish twins. Int J cancer. 2007;121:810–8. 17455257 10.1002/ijc.22746

[71] Kacprzyk LA, Laible M, Andrasiuk T, Brase JC, Börno ST, Fälth M, Kuner R, Lehrach H, Schweiger MR, Sültmann H. ERG induces epigenetic activation of Tudor domain-containing protein 1 (TDRD1) in ERG rearrangement-positive prostate cancer. PLoS ONE. 2013. 10.1371/JOURNAL.PONE.0059976. 23555854 10.1371/journal.pone.0059976 PMC3612037

[72] Lu Y, Li J, Cheng J, Lubahn DB. Messenger RNA profile analysis deciphers new Esrrb responsive genes in prostate cancer cells. BMC Mol Biol. 2015. 10.1186/S12867-015-0049-1. 26627478 10.1186/s12867-015-0049-1 PMC4667504

[73] Pham LT, Yamanaka K, Miyamoto Y, Waki H, Gouraud SSS. Estradiol-dependent gene expression profile in the amygdala of young ovariectomized spontaneously hypertensive rats. Physiol Genomics. 2022;54:99–114. 35100063 10.1152/physiolgenomics.00082.2021

[74] Yazdanpanah N, Jumentier B, Yazdanpanah M, Ong KK, Perry JRB, Manousaki D. Mendelian randomization identifies circulating proteins as biomarkers for age at menarche and age at natural menopause. Commun Biol. 2024. 10.1038/S42003-023-05737-7. 38184718 10.1038/s42003-023-05737-7 PMC10771430

[75] Kabir ER, Rahman MS, Rahman I. A review on endocrine disruptors and their possible impacts on human health. Environ Toxicol Pharmacol. 2015;40:241–58. 26164742 10.1016/j.etap.2015.06.009

[76] Lillberg K, Verkasalo PK, Kaprio J, Teppo L, Helenius H, Koskenvuo M. Stressful life events and risk of breast cancer in 10,808 women: a cohort study. Am J Epidemiol. 2003;157:415–23. 12615606 10.1093/aje/kwg002

[77] Schernhammer E, Bogl L, Hublin C, Strohmaier S, Zebrowska M, Erber A, Haghayegh S, Papantoniou K, Ollikainen M, Kaprio J. The association between night shift work and breast cancer risk in the Finnish twins cohort. Eur J Epidemiol. 2023;38:533–43. 36964875 10.1007/s10654-023-00983-9 PMC10164004

[78] Battram T, Yousefi P, Crawford G, et al. The EWAS Catalog: a database of epigenome-wide association studies. Wellcome Open Res. 2022;7:41. 35592546 10.12688/wellcomeopenres.17598.1 PMC9096146

[79] Li M, Zou D, Li Z, et al. EWAS Atlas: a curated knowledgebase of epigenome-wide association studies. Nucleic Acids Res. 2019;47:D983–8. 30364969 10.1093/nar/gky1027 PMC6324068

[80] Idelfonso-García OG, Alarcón-Sánchez BR, Vásquez-Garzón VR, Baltiérrez-Hoyos R, Villa-Treviño S, Muriel P, Serrano H, Pérez-Carreón JI, Arellanes-Robledo J. Is nucleoredoxin a master regulator of cellular redox homeostasis? Its implication in different pathologies. Antioxidants. 2022. 10.3390/ANTIOX11040670. 35453355 10.3390/antiox11040670 PMC9030443

[81] Jeong KW. Flightless I (Drosophila) homolog facilitates chromatin accessibility of the estrogen receptor α target genes in MCF-7 breast cancer cells. Biochem Biophys Res Commun. 2014;446:608–13. 24632205 10.1016/j.bbrc.2014.03.011

[82] Dou J, Schmidt RJ, Benke KS, Newschaffer C, Hertz-Picciotto I, Croen LA, Iosif AM, LaSalle JM, Fallin MD, Bakulski KM. Cord blood buffy coat DNA methylation is comparable to whole cord blood methylation. Epigenetics. 2018;13:108. 29451060 10.1080/15592294.2017.1417710 PMC5836975

[83] Ghamrawi R, Velickovic I, Milicevic O, White WM, Thistlethwaite LR, Cunningham JM, Milosavljevic A, Milic NM, Garovic VD. Buffy coat DNA methylation profile is representative of methylation patterns in white blood cell types in normal pregnancy. Front Bioeng Biotechnol. 2021;9:1. 10.3389/fbioe.2021.782843 PMC8766967 35071203

[84] Waks AG, Winer EP. Breast cancer treatment: a review. JAMA. 2019;321:288–300. 30667505 10.1001/jama.2018.19323

